# Acquisition of bipedal locomotion in a neuromusculoskeletal model with unilateral transtibial amputation

**DOI:** 10.3389/fbioe.2023.1130353

**Published:** 2023-03-01

**Authors:** Daisuke Ichimura, Hiroaki Hobara, Genki Hisano, Tsubasa Maruyama, Mitsunori Tada

**Affiliations:** ^1^ Artificial Intelligence Research Center, National Institute of Advanced Industrial Science and Technology, Tokyo, Japan; ^2^ Faculty of Advanced Engineering, Tokyo University of Science, Tokyo, Japan; ^3^ Department of Systems and Control Engineering, Tokyo Institute of Technology, Tokyo, Japan; ^4^ Research Fellow of Japan Society for the Promotion of Science (JSPS), Tokyo, Japan

**Keywords:** amputee locomotion, neuromusculoskeletal model, central pattern generator, sensory feedback, unilateral transtibial amputation, pathological locomotion

## Abstract

Adaptive locomotion is an essential behavior for animals to survive. The central pattern generator in the spinal cord is responsible for the basic rhythm of locomotion through sensory feedback coordination, resulting in energy-efficient locomotor patterns. Individuals with symmetrical body proportions exhibit an energy-efficient symmetrical gait on flat ground. In contrast, individuals with lower limb amputation, who have morphologically asymmetrical body proportions, exhibit asymmetrical gait patterns. However, it remains unclear how the nervous system adjusts the control of the lower limbs. Thus, in this study, we investigated how individuals with unilateral transtibial amputation control their left and right lower limbs during locomotion using a two-dimensional neuromusculoskeletal model. The model included a musculoskeletal model with 7 segments and 18 muscles, as well as a neural model with a central pattern generator and sensory feedback systems. Specifically, we examined whether individuals with unilateral transtibial amputation acquire prosthetic gait through a symmetric or asymmetric feedback control for the left and right lower limbs. After acquiring locomotion, the metabolic costs of transport and the symmetry of the spatiotemporal gait factors were evaluated. Regarding the metabolic costs of transportation, the symmetric control model showed values approximately twice those of the asymmetric control model, whereas both scenarios showed asymmetry of spatiotemporal gait patterns. Our results suggest that individuals with unilateral transtibial amputation can reacquire locomotion by modifying sensory feedback parameters. In particular, the model reacquired reasonable locomotion for activities of daily living by re-searching asymmetric feedback parameters for each lower limb. These results could provide insight into effective gait assessment and rehabilitation methods to reacquire locomotion in individuals with unilateral transtibial amputation.

## 1 Introduction

Locomotion is an essential behavior for animals to survive, such as to find food and to escape from threats. Several studies suggest that mammalian locomotion is controlled by a central pattern generator (CPG) in the spinal cord (Brown, 1914; Grillner and Zangger, 1975; Dimitrijevic et al., 1998). This CPG produces the basic rhythm of locomotion, which controls the flexor and extensor muscles cooperatively through the activity of motoneurons. Furthermore, the CPG is integrated with sensory feedback to achieve adaptive locomotion in various environments and body constraints (Pearson, 1995; Juvin et al., 2007), resulting in energy-efficient locomotor patterns (Hoyt and Taylor, 1981; Stenum and Choi, 2020).

Individuals with symmetrical body proportions exhibit an energy-efficient gait on flat ground (Wedge et al., 2022). The gait pattern implies symmetrical control of the left and right lower limbs by the CPG. In contrast, individuals with lower limb amputation, who have morphologically asymmetrical body proportions, exhibit asymmetrical gait patterns (Sanderson and Martin, 1997; Howard et al., 2013; Cutti et al., 2018). Previous studies reported that asymmetric gait results from various factors such as between-limb differences in muscle strength (Sibley et al., 2021), balance ability to support the body mass in the prosthetic limb (Schmid et al., 2005), and types of prostheses (Grabowski et al., 2010). These factors can lead to asymmetric gaits in individuals with lower limb amputation, which can have great variability (Howard et al., 2013) and multiple patterns (Ichimura et al., 2022) in spatiotemporal gait parameters. Such gait diversity suggests that individuals with lower limb amputation adapt their gait control patterns based on the external environment and their physical functions.

According to previous studies using split-belt treadmills, humans can implicitly adapt the spatiotemporal gait factors with sensory feedback on different speeds of the left and right belts (Reisman et al., 2005; Morton and Bastian, 2006). The adaptation was obvious in non-amputees (Darter et al., 2017), as well as in individuals with unilateral transtibial amputation (UTTA). However, the morphologically asymmetrical body proportions in individuals with UTTA inevitably result in asymmetric spatiotemporal gait parameters. Thus, clarifying the neural control of lower limbs may lead to the proposals of rehabilitation methods, duration, and strengths for individuals with lower limb amputation suffering from difficulty in acquiring locomotion (Kamrad et al., 2020).

This study aimed to investigate how individuals with UTTA control their left and right lower limbs during locomotion using a two-dimensional neuromusculoskeletal model. The forward dynamics simulation could produce physical and neural changes on the computer to enhance the understanding of human locomotion mechanisms. Various neuromusculoskeletal models have revealed the biomechanics and motor control of locomotion, such as control of human bipedal locomotion (Taga et al., 1991; Ogihara and Yamazaki, 2001; Hase and Yamazaki, 2002; Jo and Massaquoi, 2007; Aoi et al., 2010; Song and Geyer, 2015), adaptation to locomotion under pathological conditions (Ichimura and Yamazaki, 2019), and physiological characteristics of animal locomotion (Oku et al., 2021; Kim et al., 2022). Specifically, we examined whether individuals with UTTA acquire prosthetic gait through a symmetric or asymmetric feedback control for the left and right lower limbs. After acquiring locomotion, the metabolic costs of transport and the symmetry of spatiotemporal gait factors were evaluated to identify reasonable locomotion scenarios for activities of daily living in individuals with UTTA. According to a previous study, individuals with UTTA altered their gait patterns to optimize the metabolic costs of transport (Wedge et al., 2022). Therefore, we hypothesized that the asymmetric control scenario, which allows greater flexibility in gait patterns than the symmetric one, could achieve reasonable locomotion in individuals with UTTA.

## 2 Materials and methods

### 2.1 Musculoskeletal model

**FIGURE 1:**
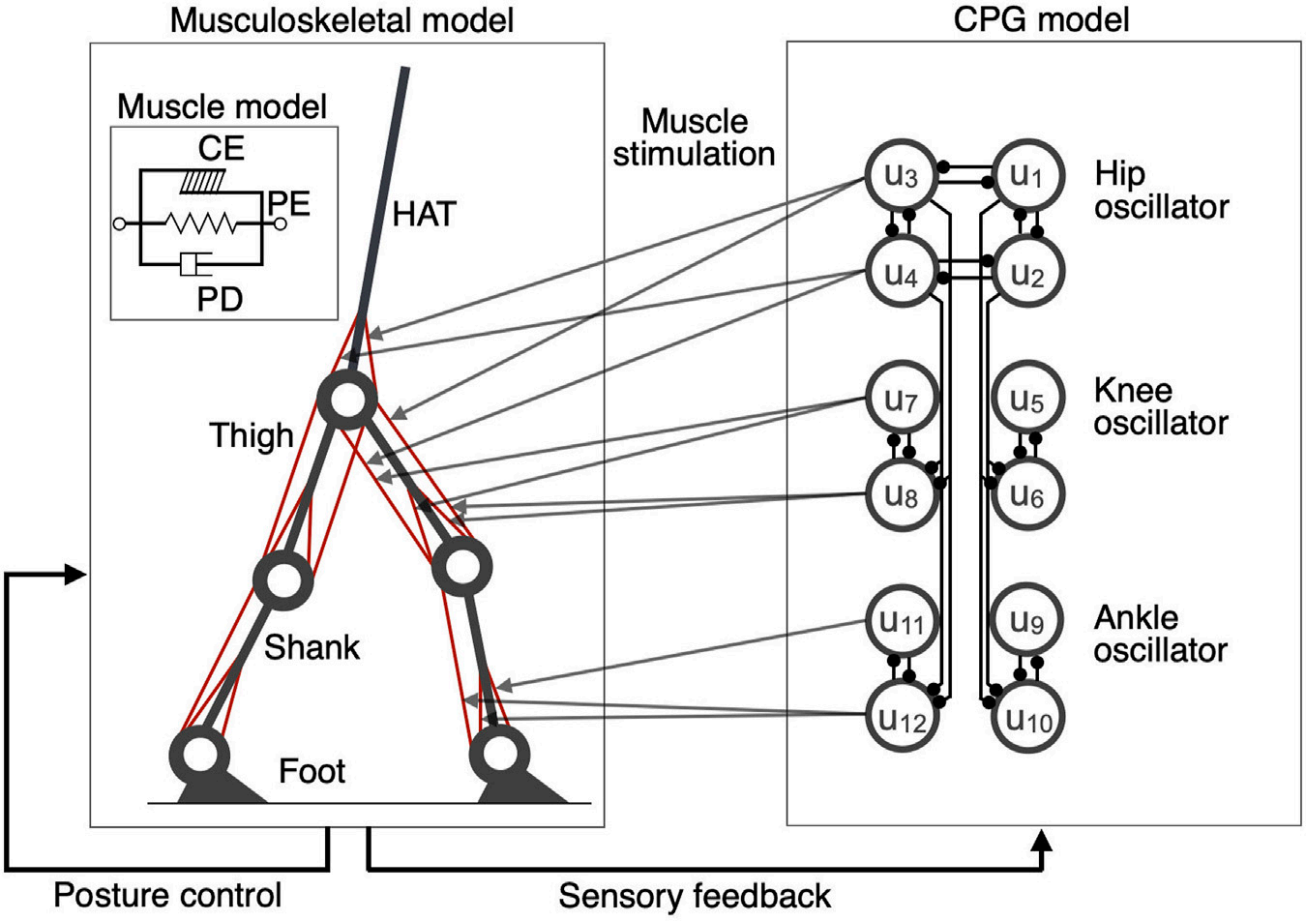
A schematic representation of the neuromusculoskeletal model. Left panel: The skeletal model consists of seven links representing the HAT (head, arms, and torso), thighs, shanks, and feet. The muscle models include (1) gluteus maximus (GM), (2) iliopsoas (IL), (3) biceps femoris long head (BFL), (4) rectus femoris (RF), (5) biceps femoris short head (BFS), (6) vastus (VA), (7) gastrocnemius (GC), (8) soleus (SO), and (9) tibialis anterior (TA). The muscle model includes contractile (CE), passive elastic (PE), and passive damping (PD) elements, respectively. Right panel: The CPG model consists of 12 internal units (*u*_1_, … , *u*_12_), generating a hip oscillator, a knee oscillator, and an ankle oscillator. The output of the CPG model corresponds to each muscle model.

Based on the results of previous studies (Ogihara and Yamazaki, 2001; Aoi et al., 2010; Ichimura and Yamazaki, 2019), we constructed a two-dimensional musculoskeletal model including the head, arms, torso (HAT), thighs, shanks, and feet (Figure 1). We determined the segment length and inertia parameters of the model based on past findings (Jo and Massaquoi, 2007; Ichimura and Yamazaki, 2019). Each joint was modeled as a pin joint with a linear viscous element. The viscosity coefficients of the hip, knee, and ankle joints were 1.09, 3.17, and 0.943 Nms/rad, respectively (Aoi et al., 2010; Ichimura and Yamazaki, 2019). The knee and ankle joints were locked to avoid hyperextension and hyperflexion which are unrealistic joint ranges of motion. The heel or toe received ground reaction forces (GRF) when they contacted the ground. The GRF is modeled by a linear spring and damper system which was employed in previous studies (Ogihara and Yamazaki, 2001; Aoi et al., 2010; Ichimura and Yamazaki, 2019) and could mimic the measured GRF. The coefficients of the spring and damper were 5.0 × 10^3^ N/m and 1.0 × 10^3^ Ns/m in the horizontal direction and 2.5 × 10^4^ N/m and 5.0 × 10^2^ Ns/m in the vertical direction, respectively (Ichimura and Yamazaki, 2019). Nine primary muscles were used in each leg for the muscle model (Figure 1): gluteus maximus (GM), iliopsoas (IL), biceps femoris long head (BFL), rectus femoris (RF), biceps femoris short head (BFS), vastus (VA), gastrocnemius (GC), soleus (SO), and tibialis anterior (TA). Muscles receive signals from the corresponding α-motoneurons and generate muscle tension through force-length and force-velocity relationships. We used the following mathematical model described by Ogihara and Yamazaki (2001), which included contractile (CE), passive elastic (PE), and passive damping (PD) elements, respectively:

$$F_{m}={\bar{F}}_{m}^{\text{CE}}\cdot k\left(\xi_{m}\right)\cdot h\left(\eta_{m}\right)\cdot \alpha_{m}+F_{m}^{\text{PD}}+F_{m}^{\text{PE}},$$

$$k\left(\xi_{m}\right)=0.32+0.71\exp\left(-1.112\left(\xi_{m}-1.0\right)\right)\sin\left(3.722\left(\xi_{m}-0.656\right)\right),$$

$$h\left(\eta_{m}\right)=1+\tanh\left(3.0\eta_{m}\right),$$

$$F_{m}^{\text{PD}}=c_{m}^{\text{PD}}{\dot{L}}_{m},$$

$$\begin{array}{c} F_{m}^{\text{PE}}=k_{m}^{\text{PE}}\left(\exp\left(15\left(L_{m}-{\bar{L}}_{m}\right)\right)-1.0\right), \end{array}$$ (1)

where $F_{m}$ is the muscle tension generated by the *m*th muscle, ${\bar{F}}_{m}^{\text{CE}}$ is the maximum muscle tension due to the CE, $k\left(\xi_{m}\right)$ is the force-length relationship, $h\left(\eta_{m}\right)$ is the force-velocity relationship, $\alpha_{m}$ is the stimulus signal from the corresponding α-motoneuron (0 ≤ $\alpha_{m}$ ≤ 1), and $F_{m}^{\text{PD}}$ and $F_{m}^{\text{PE}}$ are the forces generated by the damping and elastic elements, respectively. $\xi_{m}$ and $\eta_{m}$ are the normalized muscle length and contraction velocity divided by the muscle optimum length ${\bar{L}}_{m}$ and the muscle maximum contraction velocity ${\bar{\dot{L}}}_{m}$ , that is, where $\xi_{m}=L_{m}/{\bar{L}}_{m},\eta_{m}={\dot{L}}_{m}/{\bar{\dot{L}}}_{m}$ , $L_{m}$ , and ${\bar{\dot{L}}}_{m}$ are the muscle length and contraction velocity, respectively. $c_{m}^{\text{PD}}$ is the viscosity coefficient, and $k_{m}^{\text{PE}}$ is the coefficient of the elastic element. These parameters were also used by Ogihara and Yamazaki (2001), Aoi et al. (2010), and Ichimura and Yamazaki (2019).

### 2.2 Neural model

Generally, locomotion is considered to be generated and induced by a rhythmic neural network in the spinal cord, called CPG (Grillner and Zangger, 1975). In the present study, we used a mathematical model of the CPG (Matsuoka, 1985; Taga et al., 1991) as follows:

$$\tau_{i}\dot{u_{i}}=-u_{i}+\overset{12}{\underset{j=1}{\sum }}w_{i\;j}^{\text{CPG}}y_{j}-\beta v_{i}+u_{0}+{\text{Feed}}_{i}\left(\left\{\theta_{l}^{seg}{\}}_{l},\{GRF_{s}{\}}_{s}\;|\;\{w_{k}^{Feed}{\}}_{k}\right.\right),$$

$$\tau_{i}^{\prime }{\dot{v}}_{i}=-v_{i}+y_{i},$$

$$\begin{array}{c} y_{i}=\max\left(0,u_{i}\right), \end{array}$$ (2)

where $u_{i}$ is the internal state of the *i*th neuron, and $v_{i}$ is a variable representing the self-inhibitory effect of the *i*th neuron. $\tau_{i}$ and $\tau_{i}^{\prime }$ are time constants, β is a coefficient, and $w_{i\;j}^{\text{CPG}}$ is a connecting weight from the *j*th neuron to the *i*th neuron. $u_{0}$ is an external input, and ${\text{Feed}}_{i}$ is the feedback signal from the musculoskeletal system. $\theta_{l}^{seg}$ is the segment angle (*l* $\in \left\{\text{HAT},\text{thigh},\text{shank},\text{foot}\right\}$ for each leg), $GRF_{s}$ is the vertical GRF ( $s\in \left\{\text{left}\;\text{limb},\text{right}\;\text{limb}\right\}$ ), and $w_{k}^{Feed}$ is the weight coefficient ( k=1,⋯,32 ). The parameter values are listed in the Supplementary Material S1. $y_{i}$ also gives motor commands to α-motoneurons, which activate the muscles. The α-motoneurons also receive feedback signals from various reflexes, such as postural control. The α-motoneuron output $\alpha_{m}$ is given as follows:

$$\begin{array}{c} \alpha_{m}=\frac{2.0}{1.0+\exp\left(0.25\left(\overset{18}{\underset{i=1}{\sum }}w_{m\;i}^{\alpha }y_{i}+P_{m}\left(\left\{\theta_{j}{\}}_{j},\{\theta_{l}^{\text{seg}}{\}}_{l},\{{\text{GRF}}_{s}{\}}_{s}\;|\;\{w_{o}^{\text{POS}}{\}}_{o}\right.\right)\right)\right)}-1.0, \end{array}$$ (3)

where $w_{m\;i}^{\alpha }$ and $w_{o}^{\text{POS}}$ are the weight coefficient ( o=1,⋯,23 ), $P_{m}$ is a posture control, and $\theta_{j}$ is the joint angle (*j* $\in \{\text{hip},\text{knee},\left.\text{ankle}\right\}$ ). These parameter values are also listed in the Supplementary Material S1.

### 2.3 Generation of normal and unilateral transtibial amputation locomotion

Our model has 51 free parameters ( $u_{0}$ ; $w_{k}^{Feed}$ ; $w_{i\;j}^{\text{CPG}}$ ; $w_{o}^{\text{POS}}$ ) required to be determined to achieve stable locomotion. To search for these parameters, we employed standard genetic algorithms (GAs) (Ogihara and Yamazaki, 2001; Ichimura and Yamazaki, 2019; Oku et al., 2021). We used the evaluation function J to maximize, which is given by the following equation.

$$\begin{array}{c} J=\left\{\begin{array}{ll} D+P & \left(D<3m\right),\\ D+P+\frac{60}{C} & \left(D\geq 3m\right), \end{array}\right. \end{array}$$ (4)

$$\begin{array}{c} C=\frac{1.0}{\mathrm{TMV}}\overset{T}{\underset{t=0}{\int }}\overset{18}{\underset{m=0}{\sum }}{\dot{E}}_{m}dt, \end{array}$$ (5)

where D is the distance until the model falls, P is the penalty given when the model falls, which is set to −3.0, and C is the gross metabolic cost of transport (Koelewijn et al., 2019). T , *M*, and *V* represent the locomotion duration, body mass, and walking speed, respectively. ${\dot{E}}_{m}$ is the metabolic energy consumption by all muscles (Minetti and Alexander, 1997).

Initially, 51 free parameters were optimized using GAs to simulate normal locomotion that assumed a kinematically symmetric gait with a symmetric body (Ichimura and Yamazaki, 2019). After the model acquired normal locomotion, we simulated the pathological UTTA conditions. Specifically, the muscles of the unilateral lower leg (TA, SO, and GC) were removed. Subsequently, the weight of the lower leg, moment of inertia, and passive moment of the ankle joint were changed to 65%, 40%, and 400 Nm/rad, respectively, to mimic the lower limb prosthesis based on the findings of a previous study (Russell Esposito and Miller, 2018). The model failed to walk even for one step immediately after this manipulation. Then, we investigated two adaptation rule scenarios for this model. These scenarios optimized the parameters of the amplitude in the CPG signals and the feedback from the musculoskeletal model to the CPG model. The first scenario involved re-searching $u_{0}$ of the CPG model and the 16 symmetric feedback parameters $w_{k}^{Feed}$ for both lower limbs, which we called the ‘symmetric control model.’ This control strategy of locomotion was the same as that of the normal model. The other scenario involved re-searching $u_{0}$ of the CPG model and the 32 asymmetric feedback parameters $w_{k}^{Feed}$ for both lower limbs, which we called the ‘asymmetric control model.’ Such a control strategy for locomotion was different from that of the normal model. These scenarios assumed minimally adaptive locomotion based on the finding that the spinal cord network adapts dynamically (Rossignol et al., 2006). Finally, we analyzed the metabolic cost of transport (Eq. 5), as well as the absolute symmetry index (ASI), an indicator of the asymmetry of the spatiotemporal gait factor (Nolan et al., 2003; Bosch and Rosenbaum, 2010), as a qualitative assessment of locomotion using five steps (from the 3rd to the 8th step). The ASI was calculated by the following equation:

$$\begin{array}{c} \text{ASI}=\left|\frac{2.0\left(R-L\right)}{\left(R+L\right)}\right|\times 100, \end{array}$$ (6)

where R and L are stance time or step length of the right and left limb, respectively.

### 2.4 Implementation

We implemented GAs utilizing Message Passing Interface (MPI), which is a library for parallel computing. We wrote all programs in the C language and used the fourth-order Runge-Kutta method for the numerical solution of the differential equations. The time step size was set to 0.1 ms.

We performed five simulations with five different random number generator seeds to verify that each simulation’s results were unique. Subsequently, we observed stable bipedal locomotion (walking for continuous 10-s periods), showing no qualitative differences in locomotion patterns due to differences in the seeds.

## 3 Results

### 3.1 Generation of normal locomotion

**FIGURE 2:**
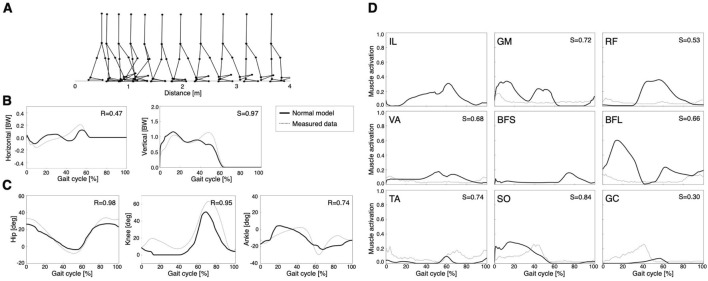
Simulation results of the normal model: **(A)** stick diagram of the normal model, **(B)** ground reaction forces, **(C)** joint angles, and **(D)** muscle activations. A gait cycle is the period of events during locomotion in which one foot contacts the ground until the same foot contacts the ground again. Dashed lines represent the measured data (Bovi et al., 2011). Solid lines indicate the right limb in the normal model. R is the correlation coefficient, and S represents cosine similarity.

The model acquired a stable locomotion after 2000 generations of GAs (Figure 2). In Figure 2A, the locomotion pattern of the model qualitatively resembles that of human bipedal locomotion. The locomotion speed of the model was 0.88 m/s. Figures 2B–D show GRFs, joint angles, and muscle activities, respectively. To evaluate the validity of the current results, the cosine similarity (S) and correlation coefficient (R) between simulation data and measured data were calculated (Bovi et al., 2011). Notably, the muscle activations of the IL and BFS were not compared with simulation data due to the lack of measurement data (Figure 2D). However, other studies demonstrated that IL activity was mainly detected in the middle of the gait cycle, whereas BFS activity mainly occurred at the end of the gait cycle, which was qualitatively consistent with the simulation results (Perry and Burnfield, 2010). Thus, normal gait in this model was validated by results that were qualitatively and quantitatively comparable with those of previous studies (Aoi et al., 2010; Song and Geyer, 2015; Aoi et al., 2019). However, the simplified model caused some differences from the human gait pattern. For example, the lack of toe joints led to smaller peaks in the latter phase of the horizontal and vertical ground forces (Figure 2B) and less ankle dorsiflexion (Figure 2C) compared to the measured data. These trends were also observed in Song and Geyer (2015).

### 3.2 Gait patterns under unilateral transtibial amputation conditions

**FIGURE 3:**
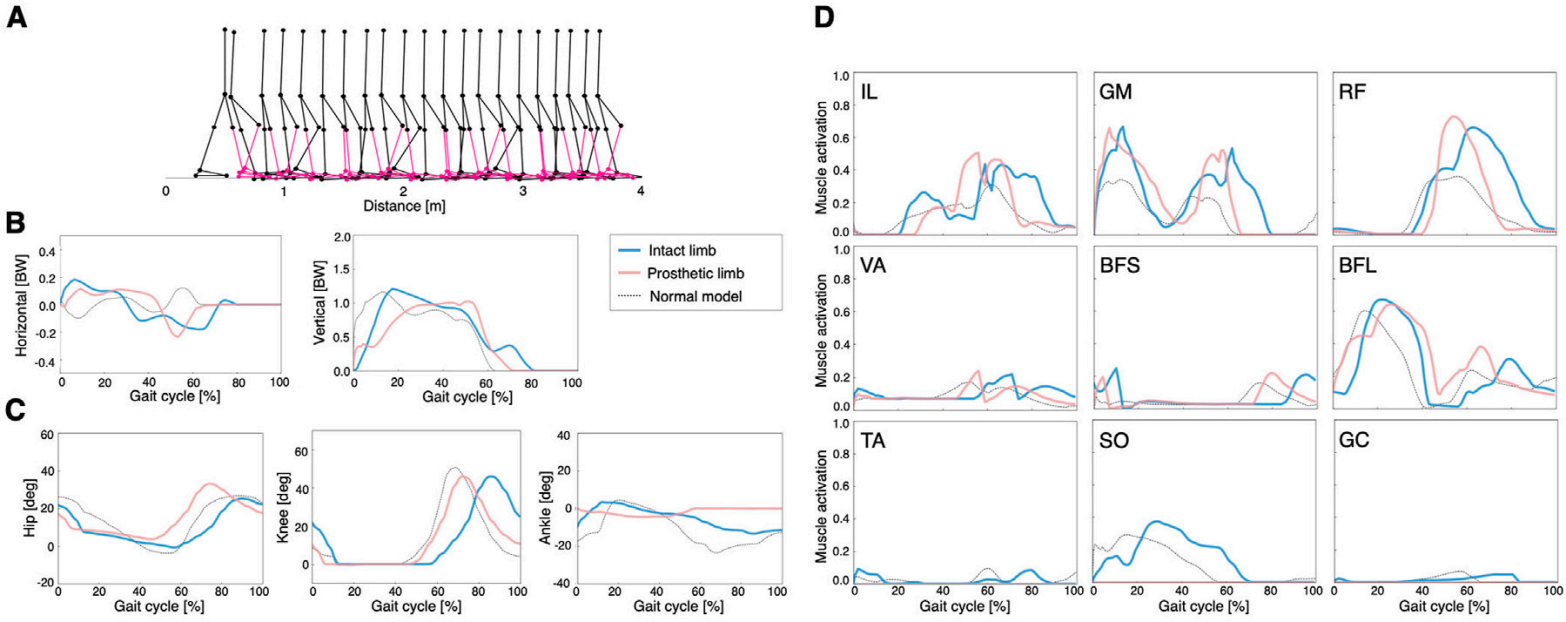
Simulation results of the symmetric control model: **(A)** stick diagram of the symmetric control model with the right ankle muscles removed and replaced by a transtibial prosthesis (red), **(B)** ground reaction forces, **(C)** joint angles, and **(D)** muscle activations. Dashed lines represent data of the normal model. Blue and red lines indicate intact and prosthetic limbs, respectively.

**FIGURE 4:**
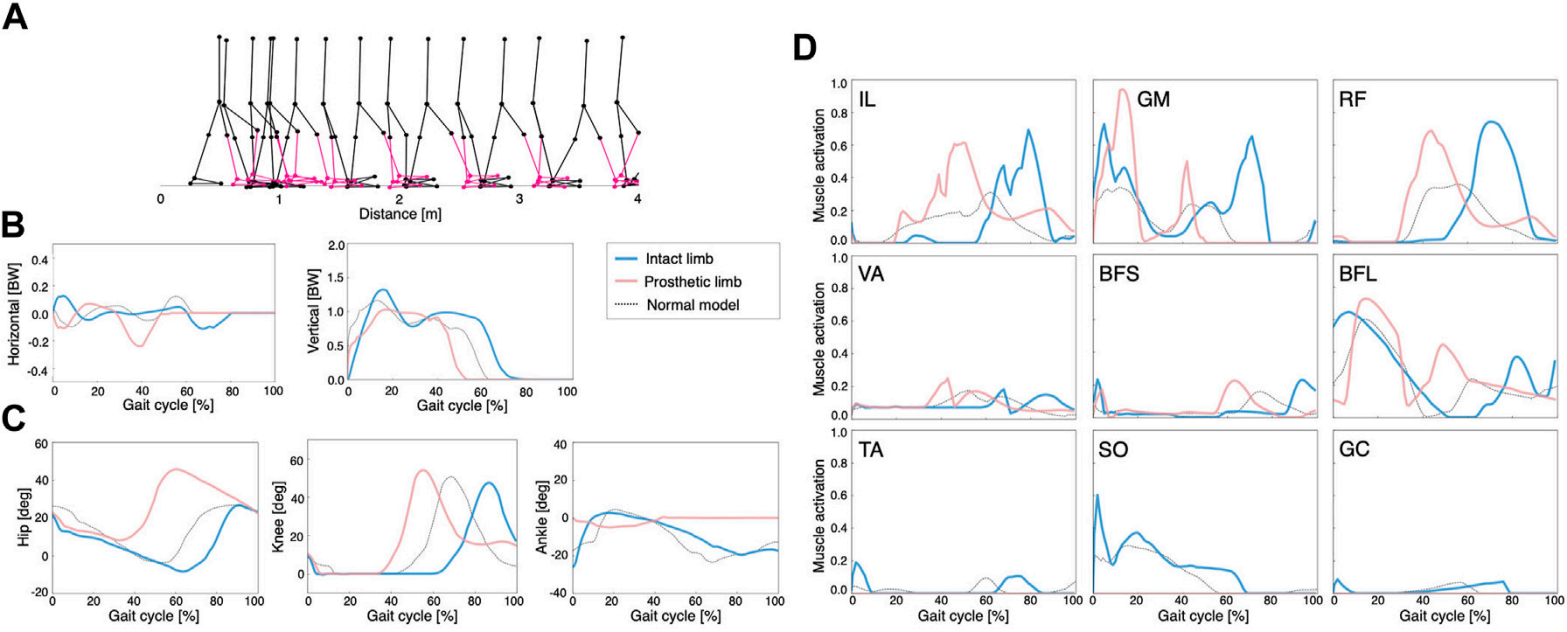
Simulation results of the asymmetric control model: **(A)** stick diagram of the asymmetric control model with the right ankle muscles removed and replaced by a transtibial prosthesis (red), **(B)** ground reaction forces, **(C)** joint angles, and **(D)** muscle activations. Dashed lines represent data of the normal model. Blue and red lines indicate intact and prosthetic limbs, respectively.

To set the UTTA condition, we attached a lower limb prosthesis to the right leg of the normal model. This manipulation caused the model to immediately fall. After 2000 generations of GAs, the symmetric control model and asymmetric control model reacquired stable locomotion, respectively (Figures 3A, 4A).

In the symmetric control model, smaller steps were observed compared to those of the other models (Figure 3A). The locomotion speed of the model was 0.32 m/s. The waveforms of GRFs, joint angles, and muscle activities were similar on the intact limb and prosthetic limb (Figures 3B–D). In addition, the peak activities of hip muscles (IL, GM, and RF) were larger than those of the other muscles, which was consistent with previous findings that hip muscle strength is related to walking ability in lower limb amputees (Nolan, 2012; Crozara et al., 2019).

In the asymmetric control model, the waveforms of GRFs, joint angles, and muscle activities differed between the intact limb and the prosthetic limb (Figures 4B–D, respectively). The locomotion speed of the model was 0.54 m/s. In addition, we observed that the peak activities of the hip muscles (IL, GM, and RF) were larger than those of the other muscles, similar to the symmetric control model. The phase space plots of these models were shown in Supplementary Material S3.

### 3.3 Comparison of metabolic costs of transport

**FIGURE 5:**
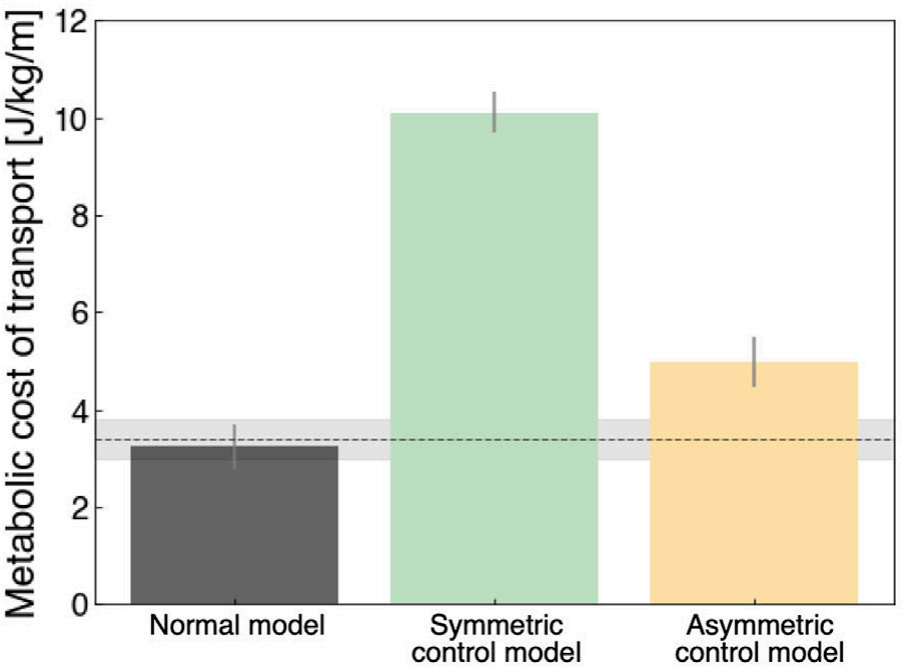
Metabolic costs of transport in each model. Each of these was calculated for 5 strides (3rd to 8th strides). Grey, green, and yellow colors indicate the normal model, symmetric control model, and asymmetric control model, respectively. The horizontal line indicates the measured data of able-bodied individuals, with a value of 3.40 ± 0.4 J/kg/m (Das Gupta et al., 2019).

Figure 5 shows the metabolic cost of transport for the qualitative evaluation of locomotion. The normal model had the lowest value (3.23 ± 0.47 J/kg/m) compared to the other models, which was approximately consistent with the findings of previous studies (Russell Esposito and Miller, 2018; Das Gupta et al., 2019; Koelewijn et al., 2019). In the UTTA condition, the symmetric control model had a value of 10.10 ± 0.42 J/kg/m, meaning approximately two times the value in the asymmetric control model (4.97 ± 0.51 J/kg/m). Previous computer simulation studies of locomotion under UTTA conditions demonstrated that the metabolic costs of transport were comparable to or less than those measured in non-amputees (Handford and Srinivasan, 2016; Russell Esposito and Miller, 2018; Miller and Russell Esposito, 2021). These studies assumed that individuals with lower limb amputation can ideally acquire kinematic gait patterns similar to those of non-amputees due to the lack of an embedded neural model. Such findings diverged from clinical practice, in which various pathological gait patterns existed (Ichimura et al., 2022). In the current study, we included a neural model in the musculoskeletal model to generate various gait patterns. Thus, the different values of metabolic costs of transport in the present study may correspond to distinct gait patterns in individuals with lower limb amputation.

### 3.4 Comparison of spatiotemporal gait patterns

**FIGURE 6:**
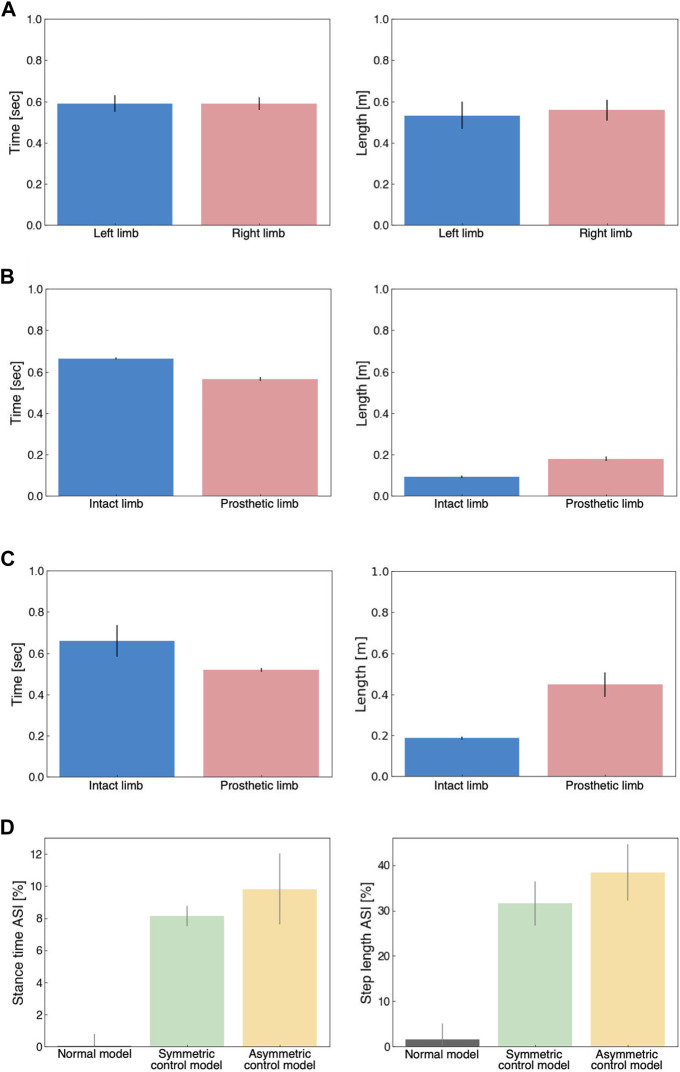
Analysis of spatiotemporal gait patterns. **(A)** Stance time (left panel) and step length (right panel) for the normal model. **(B)** Stance time (left panel) and step length (right panel) for the symmetrical control model. **(C)** Stance time (left panel) and step length (right panel) for the asymmetrical control model. **(D)** Stance time ASI (left panel) and step length ASI (right panel) for the normal model (grey), symmetric control model (green), and asymmetric control model (yellow).

Figure 6 illustrates the spatiotemporal gait patterns. In the normal model, both stance time and step length were similar in the left and right limbs (Figure 6A). Under UTTA conditions, stance time was shorter in the prosthetic limb, and step length was shorter in the intact limb (Figures 6B, C). The step length in the intact limb indicated that the intact limb was the leading limb, requiring single support in the prosthetic limb. Thus, our results suggested that prosthetic limbs in the model had weak support, which was generally consistent with measured data (Sanderson and Martin, 1997; Nolan et al., 2003). In the ASI, the UTTA condition displayed an asymmetric gait pattern, showing remarkable differences compared to the normal model (Figure 6D). However, we observed little differences in ASI between the symmetric model and the asymmetric model (stance time ASI [%]: normal model, 0.03 ± 0.76; symmetric control model, 8.14 ± 0.64; asymmetric control model, 9.83 ± 0.76; step length ASI [%]: normal model, 1.53 ± 3.60; symmetric control model, 31.67 ± 4.87; asymmetric control model, 38.37 ± 6.22). The lack of substantial differences suggested that the symmetric and asymmetric control models were difficult to distinguish based on spatiotemporal gait factors. This was consistent with previous studies indicating that functional assessment of gait ability is difficult to achieve using gait asymmetry (Hof et al., 2007; Roerdink et al., 2012).

## 4 Discussion

We implemented locomotion simulations under normal and UTTA conditions. In the normal condition, the model walked successfully after the internal parameters had been optimized by GAs. In the UTTA condition, the transtibial prosthesis was attached to the right limb of the normal model, causing walking difficulties. We investigated two adaptation rule scenarios to attempt locomotion reacquisition. The symmetric control scenario acquired stable locomotion but increased the asymmetry of the gait pattern, as well as remarkably increased metabolic costs of transport compared to the normal model. Although the asymmetric control scenario acquired stable locomotion and increased asymmetry of gait pattern, decreased metabolic costs of transport were observed, similar to the measured locomotion of individuals with UTTA (Quesada et al., 2016; Handford and Srinivasan, 2018). These results support our hypothesis that the asymmetric control scenario, which allows greater flexibility in gait patterns than the symmetric model, could achieve reasonable locomotion in individuals with UTTA.

### 4.1 Acquisition of gait under unilateral transtibial amputation conditions

The simulation of gait reacquisition for individuals with UTTA in this study was based on physiological findings, which has the validity of replicating real-world situations. Darter et al. (2017) demonstrated that individuals with UTTA are capable of adaptations to locomotion comparable to non-amputees, and Rossignol et al. (2006) showed that the spinal cord network could adapt to sensory feedback signals. In addition, motor reorganization occurs in neural circuits after lower limb amputation (Chen et al., 1998), and the neural activity of specially trained athletes, such as Paralympians, exhibited a reorganization that differs from that of able-bodied individuals (Nakazawa, 2022). Such physical changes and training could modify the control strategy of the lower limb. Based on these findings, the present study re-searched only the sensory feedback parameters to the spinal cord model (i.e., the CPG) resulting in the reacquisition of a stable gait in the UTTA condition. The results of this study suggested that the essential factors for reacquiring locomotion under pathological conditions could be found using the computer simulation.

### 4.2 Implications of symmetric or asymmetric lower limb control under unilateral transtibial amputation conditions

For activities of daily living, individuals with UTTA may require a new gait control strategy with low metabolic costs of transport, such as the asymmetric control model. How can this new behavioral control strategy be acquired? Such acquisitions of control strategies have been reported in hand rehabilitation after brain injury (Murata et al., 2008). In monkeys with brain injury, the pattern of grasping behavior changes with sufficient training, causing temporarily low grasping success rates, then markedly higher rates. In contrast, untrained monkeys could grasp, but their grasping success rates were low, and their grasping behaviors remained poor. These findings suggest that a new behavior control strategy could be acquired through sufficient training (Biernaskie et al., 2004; Sugiyama et al., 2013; Riazati et al., 2022). Similarly, in our study, the acquisition of the new gait control strategy improved the quality of locomotion in terms of low metabolic costs of transport. In other words, the symmetric control model, which is the same gait control strategy as that before the amputation, may be the initial state of training and may change to the asymmetric control model after training. Therefore, these two different gait control strategies may represent separate periods of the training process.

### 4.3 Insights for gait assessment and rehabilitation

Previous studies (Nolan, 2012; Miller et al., 2017) reported that individuals with UTTA improved their balance and walking ability after training. Most studies, however, have focused on active individuals with UTTA, which means that the factors related to walking acquisition in the early stages of training remain unclear. In addition, fewer than 20% of lower-limb amputees can walk independently (Kamrad et al., 2020), requiring investigations of this factor for locomotion acquisition. To the best of our knowledge, this is the first study to investigate such factors for locomotion acquisition in individuals with UTTA. The results of the present study suggested that differences in gait control strategies modulate locomotion qualitatively, which were difficult to identify with easily observable spatiotemporal gait factors. As reported in previous studies (Hof et al., 2007; Roerdink et al., 2012), assessments for individuals with UTTA need to be tailored towards the initial, transitional, and other periods of rehabilitation, rather than single assessments of gait asymmetry. That is, even if gait asymmetry exists during the locomotor acquisition process, such gait may represent a necessary phase and may not require forceful correction. In contrast, gait asymmetry may increase the risk of osteoarthritis (Norvell et al., 2005; Amma et al., 2021) and may require an adjustment in activity level.

### 4.4 Limitations and future works

The musculoskeletal model constructed in this study is limited to two dimensions, and the CPG model is mathematically abstracted. For example, the model would not be appropriate for detailed motion analysis in 3D space as reported by Bruel et al. (2022) or for studies of neural activity during locomotion in the cerebrum as reported by Ausborn et al. (2019). Rather, the model represents a minimal closed-loop system of human locomotion, allowing for the generation of essential behavior resulting from some operation, such as a pathological situation. In addition, we employed the same evaluation function to search the parameters in the normal and pathological condition models. For example, landing pain during walking would play a more critical role in locomotion than metabolic costs. Therefore, optimization methods for severe pathological simulation may need to be considered based on the characteristics of the disorder.

## 5 Conclusion

We constructed a musculoskeletal model equipped with a neural controller to investigate how individuals with UTTA acquire locomotion. The results of the present study suggested that individuals with UTTA can reacquire locomotion by modifying the sensory feedback parameters. In particular, the model reacquired reasonable locomotion for activities of daily living by re-searching asymmetric feedback parameters for each lower limb. These results could provide insight into effective gait assessment and rehabilitation methods to reacquire locomotion in individuals with UTTA.

## Data Availability

The original contributions presented in the study are included in the article/Supplementary Material, further inquiries can be directed to the corresponding author.
