# Supplementary material for: Acquisition of bipedal locomotion in a neuromusculoskeletal model with unilateral transtibial amputation
Source: Front Bioeng Biotechnol. 2023 Mar 1;11:1130353. doi: 10.3389/fbioe.2023.1130353 (PMC10014613; doi:10.3389/fbioe.2023.1130353)
Supplement: Supplementary file 1 [file DataSheet2.DOCX]

Supplementary Material 3

# Trajectories of each joint in phase space during bipedal locomotion

We described phase space plots of a 10 second walk in each model (Supplementary Figure 1). Each joint trajectory in each model approximates a limit cycle. The results suggest that these models achieved steady-state locomotion.


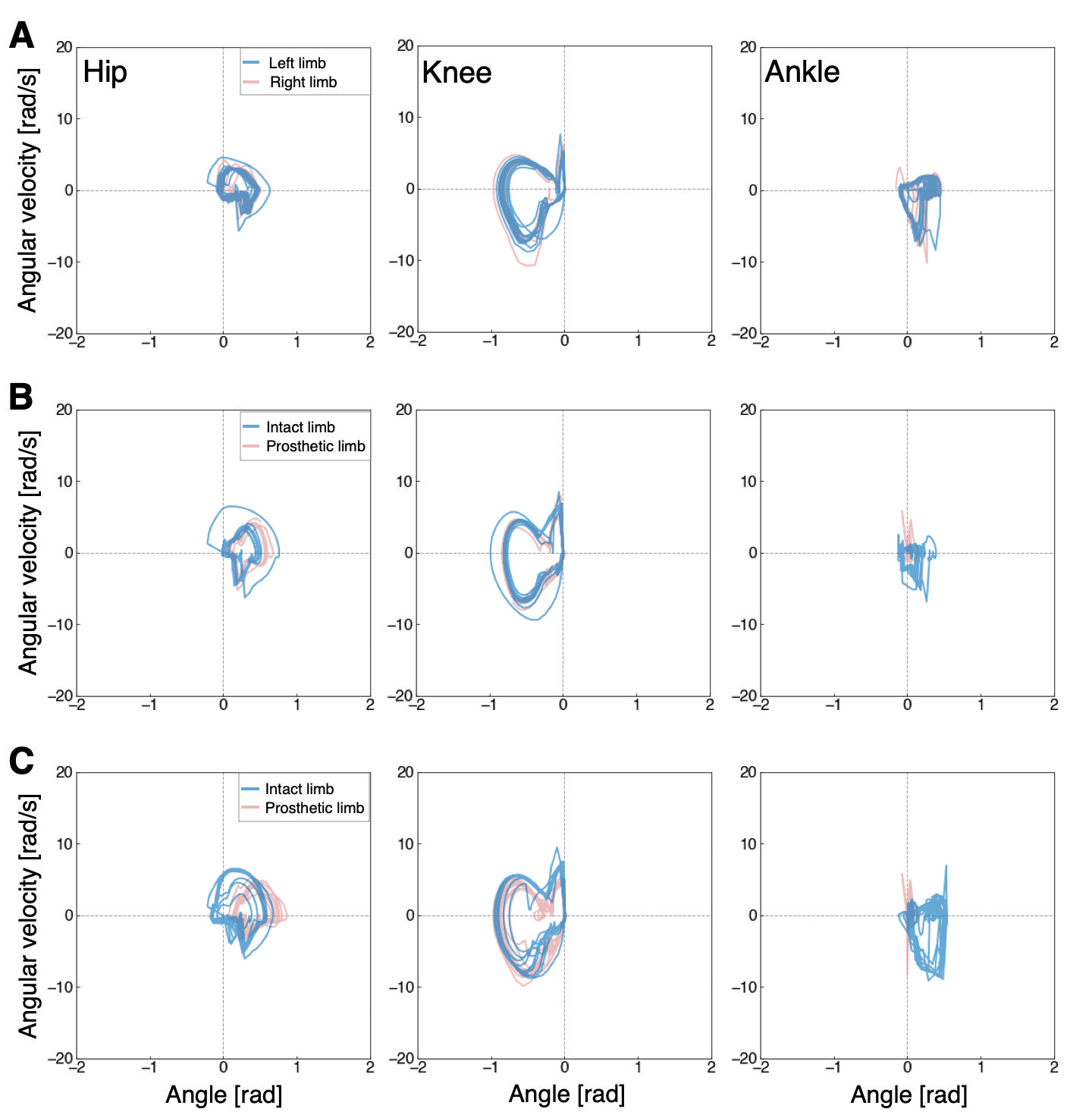


**Supplementary Figure 1.** Phase space of combinations between the angles and angular velocities in the hip, knee, and ankle joints. (A) Phase space of the normal model. Blue and red lines indicate right and left limbs, respectively. (B) Phase space of the symmetric control model. Blue and red lines indicate intact and prosthetic limbs, respectively. (C) Phase space of the asymmetric control model. Blue and red lines indicate intact and prosthetic limbs, respectively.
