# Supplementary material for: Acquisition of bipedal locomotion in a neuromusculoskeletal model with unilateral transtibial amputation
Source: Front Bioeng Biotechnol. 2023 Mar 1;11:1130353. doi: 10.3389/fbioe.2023.1130353 (PMC10014613; doi:10.3389/fbioe.2023.1130353)
Supplement: Supplementary file 3 [file DataSheet1.DOCX]

Supplementary Material

# Neural model

## Parameters for CPG model

$$\tau_{1},\ldots,\tau_{4}=0.05,\tau_{5}\ldots,\tau_{12}=0.025,\tau_{1}^{'},\ldots,\tau_{4}^{'}=0.60,\tau_{5}^{'},\ldots,\tau_{12}^{'}=0.30, \beta=2.5.$$

$$w_{i j}^{\mathrm{CPG}}= \left\{ \begin{aligned} -2.0 \left( i, j \right)\in\{ \left( 1, 2 \right), \left( 2, 1 \right), \left( 3, 4 \right), \left( 4, 3 \right), \left( 5, 6 \right), \left( 6, 5 \right), \left( 7, 8 \right), \left( 8, 7 \right), \\ \left( 9, 10 \right), \left( 10, 9 \right), \left( 11, 12 \right), (12, 11)\}, \\ -1.0 \left( i, j \right)\in\{ \left( 1, 3 \right), \left( 3, 1 \right), \left( 2, 4 \right), \left( 4, 2 \right), \left( 6, 1 \right), \left( 6, 2 \right), \left( 8, 3 \right), \left( 8, 4 \right), \\ \left( 10, 1 \right), \left( 10, 2 \right), \left( 12, 3 \right), \left( 12, 4 \right)\}, \\ 0.0 otherwise. \end{aligned} \right.$$

## Feedback signal

$$\text{Feed}_{1}=w_{1}^{\text{Feed}}\theta_{Rthigh}^{\text{seg}}-w_{2}^{\text{Feed}}\theta_{Lthigh}^{\text{seg}}+w_{3}^{\text{Feed}}\theta_{Rshank}^{\text{seg}}h\left( \text{GRF}_{R} \right)+w_{4}^{\text{Feed}}h\left( \text{GRF}_{L} \right),$$

$$\text{Feed}_{2}=-w_{5}^{\text{Feed}}\theta_{Rthigh}^{\text{seg}}+w_{6}^{\text{Feed}}\theta_{Lthigh}^{\text{seg}}-w_{7}^{\text{Feed}}\theta_{Rshank}^{\text{seg}}h\left( \text{GRF}_{R} \right)-w_{8}^{\text{Feed}}h\left( \text{GRF}_{L} \right),$$

$$\text{Feed}_{3}=w_{9}^{\text{Feed}}\theta_{Lthigh}^{\text{seg}}-w_{10}^{\text{Feed}}\theta_{Rthigh}^{\text{seg}}+w_{11}^{\text{Feed}}\theta_{Lshank}^{\text{seg}}h\left( \text{GRF}_{L} \right)+w_{12}^{\text{Feed}}h\left( \text{GRF}_{R} \right),$$

$$\text{Feed}_{4}=-w_{13}^{\text{Feed}}\theta_{Lthigh}^{\text{seg}}+w_{14}^{\text{Feed}}\theta_{Rthigh}^{\text{seg}}-w_{15}^{\text{Feed}}\theta_{Lshank}^{\text{seg}}h\left( \text{GRF}_{L} \right)-w_{16}^{\text{Feed}}h\left( \text{GRF}_{R} \right),$$

$$\text{Feed}_{5}=-w_{17}^{\text{Feed}}\theta_{Lshank}^{\text{seg}}h\left( \text{GRF}_{L} \right),$$

$$\text{Feed}_{6}=w_{18}^{\text{Feed}}\theta_{Lshank}^{\text{seg}}h\left( \text{GRF}_{L} \right),$$

$$\text{Feed}_{7}=-w_{19}^{\text{Feed}}\theta_{Rshank}^{\text{seg}}h\left( \text{GRF}_{R} \right),$$

$$\text{Feed}_{8}=w_{20}^{\text{Feed}}\theta_{Rshank}^{\text{seg}}h\left( \text{GRF}_{R} \right),$$

$$\text{Feed}_{9}=-w_{21}^{\text{Feed}}\theta_{Rshank}^{\text{seg}}h\left( \text{GRF}_{R} \right)-w_{22}^{\text{Feed}}\theta_{Lshank}^{\text{seg}}h\left( \text{GRF}_{L} \right)-w_{23}^{\text{Feed}}\dot{\theta}_{Rshank}^{\text{seg}}h\left( \text{GRF}_{R} \right),$$

$$\text{Feed}_{10}=w_{24}^{\text{Feed}}\theta_{Rshank}^{\text{seg}}h\left( \text{GRF}_{R} \right)+w_{25}^{\text{Feed}}\theta_{Lshank}^{\text{seg}}h\left( \text{GRF}_{L} \right)+w_{26}^{\text{Feed}}\dot{\theta}_{Rshank}^{\text{seg}}h\left( \text{GRF}_{R} \right),$$

$$\text{Feed}_{11}=-w_{27}^{\text{Feed}}\theta_{Lshank}^{\text{seg}}h\left( \text{GRF}_{R} \right)-w_{28}^{\text{Feed}}\theta_{Rshank}^{\text{seg}}h\left( \text{GRF}_{R} \right)-w_{29}^{\text{Feed}}\dot{\theta}_{Lshank}^{\text{seg}}h\left( \text{GRF}_{L} \right),$$

$$\text{Feed}_{12}=w_{30}^{\text{Feed}}\theta_{Lshank}^{\text{seg}}h\left( \text{GRF}_{R} \right)+w_{31}^{\text{Feed}}\theta_{Rshank}^{\text{seg}}h\left( \text{GRF}_{R} \right)+w_{32}^{\text{Feed}}\dot{\theta}_{Lshank}^{\text{seg}}h\left( \text{GRF}_{L} \right),$$

$$h\left( x \right)= \left\{ \begin{aligned} 0 \left( x\leq0 \right), \\ 1 \left( x>0 \right). \end{aligned} \right.$$

## Parameters for the normal model

$$w_{1}^{\text{Feed}}=6.533,w_{2}^{\text{Feed}}=6.196,w_{3}^{\text{Feed}}=0.150,w_{4}^{\text{Feed}}=3.120,w_{5}^{\text{Feed}}=1.271,$$

$$w_{6}^{\text{Feed}}=0.209,w_{7}^{\text{Feed}}=5.459,w_{8}^{\text{Feed}}=3.366,w_{17}^{\text{Feed}}=1.513,w_{18}^{\text{Feed}}=6.178,$$

$$w_{21}^{\text{Feed}}=2.602,w_{22}^{\text{Feed}}=25.659,w_{23}^{\text{Feed}}=1.140,w_{24}^{\text{Feed}}=8.695,w_{25}^{\text{Feed}}=0.293,$$

$$w_{26}^{\text{Feed}}=6.079.$$

Other parameters were as follows:

$$w_{9}^{\text{Feed}}=w_{1}^{\text{Feed}},w_{10}^{\text{Feed}}=w_{2}^{\text{Feed}},w_{11}^{\text{Feed}}=w_{3}^{\text{Feed}},w_{12}^{\text{Feed}}=w_{4}^{\text{Feed}},w_{13}^{\text{Feed}}=w_{5}^{\text{Feed}},$$

$$w_{14}^{\text{Feed}}=w_{6}^{\text{Feed}},w_{15}^{\text{Feed}}=w_{7}^{\text{Feed}},w_{16}^{\text{Feed}}=w_{8}^{\text{Feed}},w_{19}^{\text{Feed}}=w_{17}^{\text{Feed}},w_{20}^{\text{Feed}}=w_{18}^{\text{Feed}},$$

$$w_{27}^{\text{Feed}}=w_{21}^{\text{Feed}},w_{28}^{\text{Feed}}=w_{22}^{\text{Feed}},w_{29}^{\text{Feed}}=w_{23}^{\text{Feed}},w_{30}^{\text{Feed}}=w_{24}^{\text{Feed}},w_{31}^{\text{Feed}}=w_{25}^{\text{Feed}},$$

$$w_{32}^{\text{Feed}}=w_{26}^{\text{Feed}},u_{0}=4.890.$$

## Parameters for the symmetric control model

$$w_{1}^{\text{Feed}}=18.790,w_{2}^{\text{Feed}}=17.876,w_{3}^{\text{Feed}}=0.528,w_{4}^{\text{Feed}}=10.347,w_{5}^{\text{Feed}}=0.568,$$

$$w_{6}^{\text{Feed}}=0.800,w_{7}^{\text{Feed}}=18.396,w_{8}^{\text{Feed}}=3.141,w_{17}^{\text{Feed}}=1.171,w_{18}^{\text{Feed}}=18.700,$$

$$w_{21}^{\text{Feed}}=1.532,w_{22}^{\text{Feed}}=16.320,w_{23}^{\text{Feed}}=2.109,w_{24}^{\text{Feed}}=24.156,w_{25}^{\text{Feed}}=0.564,$$

$$w_{26}^{\text{Feed}}=7.702.$$

Other parameters were as follows:

$$w_{9}^{\text{Feed}}=w_{1}^{\text{Feed}},w_{10}^{\text{Feed}}=w_{2}^{\text{Feed}},w_{11}^{\text{Feed}}=w_{3}^{\text{Feed}},w_{12}^{\text{Feed}}=w_{4}^{\text{Feed}},w_{13}^{\text{Feed}}=w_{5}^{\text{Feed}},$$

$$w_{14}^{\text{Feed}}=w_{6}^{\text{Feed}},w_{15}^{\text{Feed}}=w_{7}^{\text{Feed}},w_{16}^{\text{Feed}}=w_{8}^{\text{Feed}},w_{19}^{\text{Feed}}=w_{17}^{\text{Feed}},w_{20}^{\text{Feed}}=w_{18}^{\text{Feed}},$$

$$w_{27}^{\text{Feed}}=w_{21}^{\text{Feed}},w_{28}^{\text{Feed}}=w_{22}^{\text{Feed}},w_{29}^{\text{Feed}}=w_{23}^{\text{Feed}},w_{30}^{\text{Feed}}=w_{24}^{\text{Feed}},w_{31}^{\text{Feed}}=w_{25}^{\text{Feed}},$$

$$w_{32}^{\text{Feed}}=w_{26}^{\text{Feed}},u_{0}=5.589.$$

## Parameters for the asymmetric control model

$$w_{1}^{\text{Feed}}=12.570,w_{2}^{\text{Feed}}=19.952,w_{3}^{\text{Feed}}=0.437,w_{4}^{\text{Feed}}=12.310,w_{5}^{\text{Feed}}=1.736,$$

$$w_{6}^{\text{Feed}}=0.777,w_{7}^{\text{Feed}}=12.560,w_{8}^{\text{Feed}}=7.504,w_{9}^{\text{Feed}}=22.279,w_{10}^{\text{Feed}}=0.651,$$

$$w_{11}^{\text{Feed}}=0.257,w_{12}^{\text{Feed}}=11.789,w_{13}^{\text{Feed}}=3.961,w_{14}^{\text{Feed}}=0.038,w_{15}^{\text{Feed}}=8.794,$$

$$w_{16}^{\text{Feed}}=0.454,w_{17}^{\text{Feed}}=4.820,w_{18}^{\text{Feed}}=24.335,w_{19}^{\text{Feed}}=3.962,w_{20}^{\text{Feed}}=21.894,$$

$$w_{21}^{\text{Feed}}=5.638,w_{22}^{\text{Feed}}=46.367,w_{23}^{\text{Feed}}=1.646,w_{24}^{\text{Feed}}=14.200,w_{25}^{\text{Feed}}=0.686,$$

$$w_{26}^{\text{Feed}}=10.694,w_{27}^{\text{Feed}}=5.734,w_{28}^{\text{Feed}}=46.444,w_{29}^{\text{Feed}}=1.501,w_{30}^{\text{Feed}}=25.817,$$

$$w_{31}^{\text{Feed}}=0.098,w_{32}^{\text{Feed}}=2.304 ,u_{0}=4.153.$$

# α-motor neuron model

## Posture control

$$\text{P}_{Rbfl}=w_{1}^{\text{POS}}\theta_{Rhip}+w_{2}^{\text{POS}}f\left( \dot{\theta}_{Rhip} \right),$$

$$\text{P}_{Lbfl}=w_{1}^{\text{POS}}\theta_{Lhip}+w_{2}^{\text{POS}}f\left( \dot{\theta}_{Lhip} \right),$$

$$\text{P}_{Rbfs}=w_{3}^{\text{POS}}f\left( \dot{\theta}_{Rknee} \right),$$

$$\text{P}_{Lbfs}=w_{3}^{\text{POS}}f\left( \dot{\theta}_{Lknee} \right),$$

$$\text{P}_{Rva}=-w_{4}^{\text{POS}}\theta_{Rknee}-w_{5}^{\text{POS}}f\left( \dot{\theta}_{Rknee} \right)h\left( \text{GRF}_{R} \right)+w_{6}^{\text{POS}}h\left( \text{GRF}_{R} \right),$$

$$\text{P}_{Lva}=-w_{4}^{\text{POS}}\theta_{Lknee}-w_{5}^{\text{POS}}f\left( \dot{\theta}_{Lknee} \right)h\left( \text{GRF}_{L} \right)+w_{6}^{\text{POS}}h\left( \text{GRF}_{L} \right),$$

$$\text{P}_{Ril}=-w_{7}^{\text{POS}}\theta_{HAT}^{\text{seg}}-w_{8}^{\text{POS}}\dot{\theta}_{HAT}^{\text{seg}}h\left( \text{GRF}_{R} \right)-w_{9}^{\text{POS}}f\left( \theta_{Rhip} \right)-w_{10}^{\text{POS}}f\left( \dot{\theta}_{Rhip} \right),$$

$$\text{P}_{Lil}=-w_{7}^{\text{POS}}\theta_{HAT}^{\text{seg}}-w_{8}^{\text{POS}}\dot{\theta}_{HAT}^{\text{seg}}h\left( \text{GRF}_{L} \right)-w_{9}^{\text{POS}}f\left( \theta_{Lhip} \right)-w_{10}^{\text{POS}}f\left( \dot{\theta}_{Lhip} \right),$$

$$\text{P}_{Rgm}=w_{11}^{\text{POS}}\theta_{HAT}^{\text{seg}}+w_{12}^{\text{POS}}\dot{\theta}_{HAT}^{\text{seg}}h\left( \text{GRF}_{R} \right)+w_{13}^{\text{POS}}h\left( \text{GRF}_{R} \right),$$

$$\text{P}_{Lgm}=w_{11}^{\text{POS}}\theta_{HAT}^{\text{seg}}+w_{12}^{\text{POS}}\dot{\theta}_{HAT}^{\text{seg}}h\left( \text{GRF}_{L} \right)+w_{13}^{\text{POS}}h\left( \text{GRF}_{L} \right),$$

$$\text{P}_{Rrf}=-w_{14}^{\text{POS}}f\left( \theta_{Rknee} \right)-w_{15}^{\text{POS}}f\left( \theta_{Rhip} \right)h\left( \text{GRF}_{R} \right)+w_{16}^{\text{POS}}h\left( \text{GRF}_{R} \right),$$

$$\text{P}_{Lrf}=-w_{14}^{\text{POS}}f\left( \theta_{Lknee} \right)-w_{15}^{\text{POS}}f\left( \theta_{Lhip} \right)h\left( \text{GRF}_{L} \right)+w_{16}^{\text{POS}}h\left( \text{GRF}_{L} \right),$$

$$\text{P}_{Rta}=w_{17}^{\text{POS}}\dot{\theta}_{Rfoot}^{\text{seg}}h\left( \text{GRF}_{L} \right),$$

$$\text{P}_{Lta}=w_{17}^{\text{POS}}\dot{\theta}_{Lfoot}^{\text{seg}}h\left( \text{GRF}_{R} \right),$$

$$\text{P}_{Rgc}=w_{18}^{\text{POS}}f\left( \theta_{Rankle} \right)h\left( \text{GRF}_{R} \right)+w_{19}^{\text{POS}}f\left( \text{GRF}_{R} \right),$$

$$\text{P}_{Lgc}=w_{18}^{\text{POS}}f\left( \theta_{Lankle} \right)h\left( \text{GRF}_{L} \right)+w_{19}^{\text{POS}}f\left( \text{GRF}_{L} \right),$$

$$\text{P}_{Rso}=-w_{20}^{\text{POS}}f\left( \theta_{Rfoot}^{\text{seg}} \right)h\left( \text{GRF}_{R} \right)-w_{21}^{\text{POS}}\dot{\theta}_{Rfoot}^{\text{seg}}h\left( \text{GRF}_{R} \right)+w_{22}^{\text{POS}}\theta_{Rankle}h\left( \text{GRF}_{R} \right)+w_{23}^{\text{POS}}f\left( \text{GRF}_{R} \right),$$

$$\text{P}_{Lso}=-w_{20}^{\text{POS}}f\left( \theta_{Lfoot}^{\text{seg}} \right)h\left( \text{GRF}_{L} \right)-w_{21}^{\text{POS}}\dot{\theta}_{Lfoot}^{\text{seg}}h\left( \text{GRF}_{L} \right)+w_{22}^{\text{POS}}\theta_{Lankle}h\left( \text{GRF}_{L} \right)+w_{23}^{\text{POS}}f\left( \text{GRF}_{L} \right),$$

$$f=\text{max}\left( 0,x \right).$$

## Parameters for α-motor neuron

$$w_{bfl 1}^{\alpha}=0.068,w_{bfl 2}^{\alpha}=1.428,w_{bfs 1}^{\alpha}=0.128,w_{va 1}^{\alpha}=0.001,w_{il 1}^{\alpha}=0.783,$$

$$w_{gm 1}^{\alpha}=0.798,w_{rf 1}^{\alpha}=0.604,w_{rf 2}^{\alpha}=0.046,w_{ta 1}^{\alpha}=0.101,w_{gc 1}^{\alpha}=0.003,$$

$$w_{so 1}^{\alpha}=0.089,$$

$$\text{otherwise} w_{m i}^{\alpha}=0.0.$$

$$w_{1}^{\text{POS}}=2.152,w_{2}^{\text{POS}}=0.564,w_{3}^{\text{POS}}=0.341,w_{4}^{\text{POS}}=1.440,w_{5}^{\text{POS}}=0.288,$$

$$w_{6}^{\text{POS}}=0.552,w_{7}^{\text{POS}}=92.146,w_{8}^{\text{POS}}=31.456,w_{9}^{\text{POS}}=4.937,w_{10}^{\text{POS}}=0.344,$$

$$w_{11}^{\text{POS}}=47.583,w_{12}^{\text{POS}}=30.428,w_{13}^{\text{POS}}=0.049,w_{14}^{\text{POS}}=0.368,w_{15}^{\text{POS}}=0.930,$$

$$w_{16}^{\text{POS}}=0.032,w_{17}^{\text{POS}}=0.182,w_{18}^{\text{POS}}=1.537,w_{19}^{\text{POS}}=0.001,w_{20}^{\text{POS}}=36.408,$$

$$w_{21}^{\text{POS}}=2.018,w_{22}^{\text{POS}}=0.150,w_{23}^{\text{POS}}=0.001.$$
